# Modular framework to assess the risk of African swine fever virus entry into the European Union

**DOI:** 10.1186/1746-6148-10-145

**Published:** 2014-07-03

**Authors:** Lina Mur, Beatriz Martínez-López, Solenne Costard, Ana de la Torre, Bryony A Jones, Marta Martínez, Fernando Sánchez-Vizcaíno, María Jesús Muñoz, Dirk U Pfeiffer, José Manuel Sánchez-Vizcaíno, Barbara Wieland

**Affiliations:** 1 VISAVET Center and Animal Health Department, Veterinary School, Universidad Complutense de Madrid, Avenida Puerta de Hierro s/n, 28040 Madrid, Spain; 2 Veterinary Epidemiology, Economics & Public Health Group, Royal Veterinary College, Hawkshead Lane, North Mymms, Hatfield, Hertfordshire AL9 7TA, UK; 3 Research Centre in Animal Health, CISA/INIA, Carretera de Algete a El Casar s/n, 28130 Valdeolmos, Madrid, Spain; 4 Center of Animal Disease Modeling and Surveillance (CADMS), University of California, Davis, USA; 5 Swiss Agency for Development and Cooperation, Ulaanbataar, Mongolia; 6 EpiX Analytics, 1643 Spruce Street, Boulder 80302, CO, USA

**Keywords:** African swine fever, Emerging disease, Introduction, European Union, Pigs, Risk assessment, Semi-quantitative framework, Transboundary disease

## Abstract

**Background:**

The recent occurrence and spread of African swine fever (ASF) in Eastern Europe is perceived as a serious risk for the pig industry in the European Union (EU). In order to estimate the potential risk of ASF virus (ASFV) entering the EU, several pathways of introduction were previously assessed separately. The present work aimed to integrate five of these assessments (legal imports of pigs, legal imports of products, illegal imports of products, fomites associated with transport and wild boar movements) into a modular tool that facilitates the visualization and comprehension of the relative risk of ASFV introduction into the EU by each analyzed pathway.

**Results:**

The framework’s results indicate that 48% of EU countries are at relatively high risk (risk score 4 or 5 out of 5) for ASFV entry for at least one analyzed pathway. Four of these countries obtained the maximum risk score for one pathway: Bulgaria for legally imported products during the high risk period (HRP); Finland for wild boar; Slovenia and Sweden for legally imported pigs during the HRP. Distribution of risk considerably differed from one pathway to another; for some pathways, the risk was concentrated in a few countries (e.g., transport fomites), whereas other pathways incurred a high risk for 4 or 5 countries (legal pigs, illegal imports and wild boar).

**Conclusions:**

The modular framework, developed to estimate the risk of ASFV entry into the EU, is available in a public domain, and is a transparent, easy-to-interpret tool that can be updated and adapted if required. The model’s results determine the EU countries at higher risk for each ASFV introduction route, and provide a useful basis to develop a global coordinated program to improve ASFV prevention in the EU.

## Background

The European Union (EU) has an ever-increasingly highly industrialized and specialized pig production sector [1], and is the second largest pig producer in the world with 22.6 million tons of pork produced in 2012 [2]. To maintain this high level of production and the current swine health status, it is crucial to prevent the introduction and re-introduction of infectious diseases, particularly OIE notifiable diseases such as African swine fever (ASF). The introduction of ASF into any EU country would result in an immediate export ban of pigs and pig products from the infected area. This could cause huge losses for the affected country, especially given the ban of intra-EU movements as it poses the highest volume of trade, but also to the EU as a whole. The EU is a net exporter of pork, with 2.3 million tons of pork exported to third countries in 2012. Furthermore, there is the potential to increase exports due to high demand from Russia and China [2], besides important intra-EU trade.

ASF is one of the most devastating swine diseases given its high mortality, economic losses as a result of trade restrictions, and the fact that no vaccine is available for its control [3]. Traditionally, this viral infectious disease has been widespread on the African continent, where it still remains in many countries. In 1960, ASF spread to some southern European countries (Portugal and Spain), where it persisted for more than 30 years [4]. On the island of Sardinia (Italy), ASF has been endemic since 1978, which is the cause of much concern, although it has not spread to other EU areas [5]. In other European countries and some territories of the American continent (e.g., Brazil [6], the Dominican Republic, Haiti and Cuba [7]), ASF virus (ASFV) was also introduced in the 20th century, but was swiftly eradicated by drastic control programs.

Since its introduction into Georgia in 2007 [8], ASFV has spread to Russia, Armenia, Azerbaijan, and later to the Ukraine and Belarus, and has affected both domestic pig and wild boar (*Sus scrofa* L.) populations. More recently, in January and February 2014, ASF was confirmed in dead wild boars in Lithuania and Poland [9]. This situation is perceived to be a major threat for European pig producers and has increased disease awareness among Eastern EU member states [10,11].

Since there is no vaccine available for ASFV, disease prevention and rapid control require safe disposal of waste from international ports and airports, effective surveillance, disease awareness in high-risk areas, and regularly updated control and contingency plans. The design of tailored prevention and control strategies benefits from knowledge of the identification and allocation of the risks for ASFV introduction, which can be generated transparently through risk assessment [12].

Various EU countries have already made country-specific risk assessments. Finland has analyzed the potential routes of ASFV introduction by emphasizing the risk of wild boar infection in the country, as well as the importance of biosecurity measures to prevent domestic pigs coming into contact with ASFV [13]. Germany has analyzed the risk associated with returning animal transport vehicles, which it has classified as moderate for the transport of breeding pigs, but as high for movements of pigs for fattening and slaughter [14]. Other analyses have been carried out to estimate the risk of ASFV introduction into the United Kingdom [15], Poland [16] and Denmark [17]. However, none has been published in the peer-reviewed scientific literature. Consequently, the results are available only upon request to the authors.

The risk of ASFV entry into the EU has been analyzed by the authors of the present study for five different pathways, these being: i) legal imports of live pigs during the high-risk period (HRP; i.e. before detecting the first case in the exporting country) [18]; ii) legal imports of different types of pig products also during the HRP [19]; iii) illegal imports of pig products [20]; iv) transport fomites (including contaminated trucks or waste from international planes and ships) [21]; and v) wild boar movements [22]. The objective of the present work was to integrate all these pathways into a flexible and transparent modular framework to enable the visualization and comprehension of the risk of ASFV introduction into EU by these five different pathways. The results of this framework will identify the EU countries at higher risk for all the analyzed pathways, in which specific control strategies can be adopted to prevent ASFV entry into the EU. In addition, the provided structure can be later used as an example to assess the risk of ASF entrance into other regions or can be conveniently adapted for the introduction of other diseases.

## Methods

### Model structure

**Figure 1:**
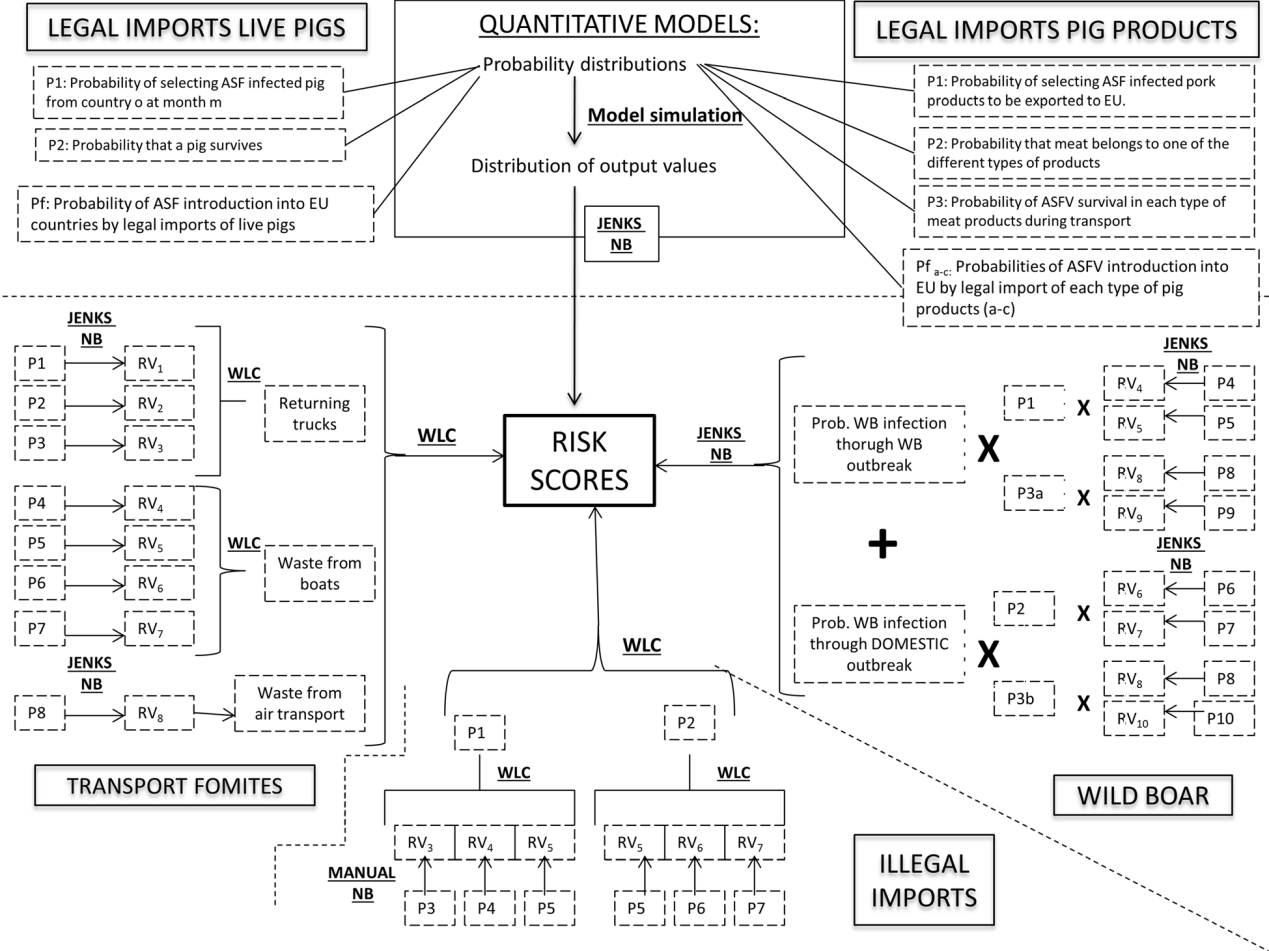
**Detailed structure of the modular framework.** The five risk pathway modules are represented and include the main steps of the respective quantitative and semi-quantitative models. P (Probability in the quantitative assessments, Proxy in the semi-quantitative assessments), RV (Risk Value), JENKS NB (Jenks Natural Breaks), WLC (Weighted linear combination of values), Manual NB (Manual Natural Breaks), X (Multiplication of values) + (sum of values).

A modular risk assessment framework was developed to separately integrate the risk assessments for the five main routes of entry (Figure 1) in order to provide an overview of the risk of ASF introduction into the EU. Five modules were developed to estimate the risk of ASFV entry into 27 of the 28 EU countries^a^ via each analyzed pathway: legal imports of live pigs, legal imports of pig products, illegal imports of pig products, transport-associated fomites, and wild boar movements. The risk pathways included in the analysis were based on a literature review that considers ASFV transmission mechanisms, known routes of introduction into previously free areas, and the current epidemiological situation.

All five modules, and consequently the modular framework that integrates them, correspond to the entry assessment component as defined in the OIE risk analysis framework, where entry addresses the likelihood of a commodity infected by a certain pathogen (hazard) being released into a particular territory [12]. The exposure and consequence assessments, which have been previously published [23], were not included.

### Description of pathway modules

Different approaches were taken to develop the five risk assessment modules after considering pathway-specific characteristics and data availability. The risk of entry via legal import pathways was estimated by quantitative models, whereas risk was assessed by semi-quantitative models for illegal imports, wild boar and transport-associated fomites.

The legal import (live pigs and pig products) risk assessments provided risk estimates as probability distributions for ASFV introduction. In order to facilitate the visualization and interpretation of these results, the mean probabilities of ASFV introduction were categorized into six risk scores (risk scores from 0 to 5) based on natural breaks calculated by Jenks algorithm [24]. All the semi-quantitative models followed a similar approach using proxy indicators (parameters related to the level of risk and for which enough data are available). The data values for each proxy indicator were categorized using natural breaks (by Jenks algorithm for transport-associated fomites and wild boar, and manual natural breaks for the illegal pathway), and were consequently converted into six scores (scores from 0 to 5). Each module produced an overall risk score for all the EU member states, calculated by a weighted linear combination of the scores of the proxy indicators. The weights of each parameter were obtained by expert opinion elicitation. The use of such methodology produces risk scores from 0 to 5 that reflect probabilities of 0 = negligible, 1 = very low, 2 = low, 3 = medium, 4 = high and 5 = very high to compare the remaining countries within the same pathway.

The detailed structure, parameters, inputs and outputs for these modules are available in separate publications [18-22]. The present work provides only a brief summary of each module in order to facilitate the comprehension of the adjustments made and, consequently, the proper interpretation of the results obtained.

#### Module 1 and 2: Legal imports

**Figure 2:**
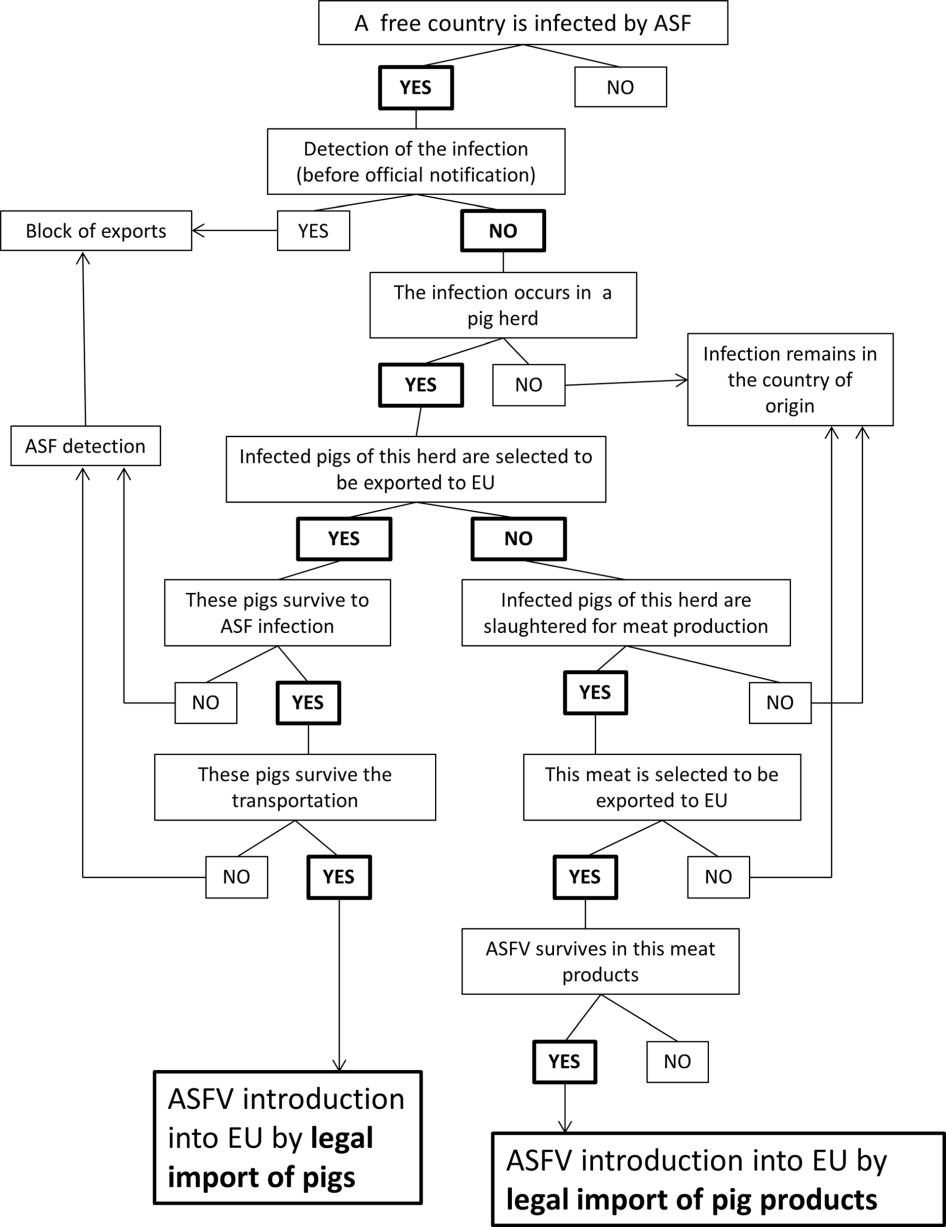
Scenario tree for the legal import of pigs and legal imports of products pathways.

For both legal import pathways (i.e., live pigs and pig products), two quantitative risk assessments [18,19] were conducted as detailed data were available for not only the frequencies and amounts of imports of pigs and products from extra-EU countries (considered to be countries of origin, or “country o”) to EU member states (countries of destination “d”), but also for the numbers of pigs and pork production quantities in the countries of origin [25]. For both modules, a similar structure based on a scenario tree was used (Figure 2). For pig products, three different types of pork products, referred to as fresh meat (referred to as “a”), frozen meat (referred to as “b”) and processed products (salted, smoked or fat products) (referred to as “c”), were considered to account for the different ASFV survival periods for these products [19]. The models were developed in @ Risk version 5.5 (Palisade Corporation, Newfield, NY, USA).

#### Module 3: Illegal imports of pig products

As data on illegal imports are very scarce and the probabilities associated with most steps of the risk pathway would be difficult to estimate quantitatively, a different approach was adopted. Illegal importation of pig products for personal consumption and for commercial purposes was modeled semi-quantitatively using ten social, economic and geographical factors, also called proxy indicators, which were assumed to influence the risk of illegal import (more information is available in Table 1). Further details on the illegal import model and its results are available in [20].

**Table 1:** Parameters and sources of data employed in the modular framework for the ASF risk assessment

**Pathway**	**Parameter**	**Definition**	**Source**
L.PIGS &L.PROD	Po	Probability of infection in the country of origin (country o)	Beta(α_1_, α_2_)
			α_1_ = X + 1; α2 = M-(X + 1)
			X:number of outbreaks by month[26]
			M: number of months considered
	Ou	Number of undetected outbreaks before official notification in country o	[9]
	To	Average herd size in country o	To = No/So
	No	Pig population in country o	[9,27]
	So	Pig establishments in country o	[27,28]
	Hp	Intra-herd prevalence	[29,30]
	Ps	Probability of an ASF-infected pig surviving infection	[31]
L.PIGS	P1s	Probability of selecting an ASF-infected pig from country o in month m	Beta (α_1_, α_2_) α_1_ = NI + 1; α2 = No-(NI + 1) NI = Po x Ou x To x Hp;
	Pt	Probability of survival during transportation	[32]
	P2_S_	Probability of a pig surviving	P2_S_ = Ps*Pt
	Sn^odm^	Imports of live swine (number of pigs) from country of origin (o) to the EU destination country (d) in month m (in the last 5 years). In order to transform Eurostat imports data (in 100 kg) into number of pigs, a standard weight of 100 kg was assumed per pig.	[25]
			Normal (μ, σ)
	Sp_odm_	Probability of an ASF infected animal from country o entering country d in month (m)	Binomial (n, p)
			n = Sn^odm^
			p = P1_S_ x P2_S_
	Pf_S_	Probability of having at least one introduction of ASFV into one EU country (d) from one of country of origin (o) in month m by legal imports of live pigs	$\mathrm{Pfs}=1-\prod_{\mathrm{odm}}\left(1-sp_{\mathrm{odm}}\right)$
	Pm	Probability of a pig being grown for meat production	Normal(Mo/No)
L. PRODS	Mo	Number of slaughtered pigs in countries o	[27] (2005/2009)
	PC	kg of meat obtained per slaughtered pig (per 100 kg)	[27] (2000-2009)
	Co	Annual pig meat production per country (100 kg)	[27] (2005-2008)
	P1_P_	Probability of selecting infected ASFV pig meat from country o in month m	P1p: Mi / (Co/12),
			Mi: Po*Ou*To*Hp*Ps*Pm*PC
	P2_P_	Probability of meat belonging to one of the different types of products considered	[25] (2008-2009)
	P3_P_	Probability of ASF virus survival in each meat product type during transport.	[33,34]
	pn^odm^	Imports of each pig meat product type (100 kg weight) from country “o” to EU country “d” in month “m”	[25] (2005-2009)
	Pp_odm_	Probabilities of ASF infected pig products of different types (a-c) from country o entering country d in month m	Binomial (n, p)
			n = pn^odm^
			p = P1_p_* P2_p_ * P3_p_
	Pf_p_	Probabilities of having at least one introduction of ASFV into one EU country (d) from one country (o) in month m by legal imports of each pig products type (a-c).	$\mathrm{Pfp}=1-\prod_{\mathrm{odm}}\left(1-Pp_{\mathrm{odm}}\right)$
	PT_P_	Probabilities of having at least one introduction of ASFV into one EU country (d) from one country o in month m by legal imports of any pig product type.	$\mathrm{PTp}=1-\prod_{i=a}^{c}\left(1-Pf_{p}\right)$
ILLEGAL	P1_I_	Probability of release through illegal importation for personal consumption	Sum of weighted risk scores for P3, P4 and P5
	P3_I_	Outbound tourism to ASF-affected countries. Holiday or business trips of 1 night or more from EC27 to Africa and Russia, arrivals of non resident visitors at national borders of Georgia	[25], Georgian National Tourism Agency
	P4_I_	Inbound tourism from ASF-affected countries: Arrivals to EU27 of non residents from Africa and Russia staying in hotels, etc.	[25]
	P5 _I_	Residents (citizens )from ASF-affected countries	[25]
	P2 _I_	Probability of release through illegal importation for commercial purposes	Sum of weighted risk scores for P5, P6 and P7
	P6_I_	Price of pork. 2010 annual average price of Grade E carcasses (55-59% lean meat percentage) in euros per 100 kg	European community
	P7_I_	Geographic position	Sum of weighted risk scores (P8,P9,P10)
	P8_I_	Number of ports and airports	World Port Index 2009; [25]
	P9_I_	Distance in km to nearest ASF-affected country (from country border to border of nearest ASF-affected country)	Shapefile of national boundaries
	P10_I_	Number of international terrestrial border points with non EU member states	FAO Geonetwork: shapefiles of railways, roads and waterways of the World VMAP)
TAF	P1t	Number of potential ASF-contaminated returning trucks. Number of live pigs exported from EU to ASF-affected countries by road	[25] (Nov. 2007-2009)- (ComExt)
	P2t	Number of ways (and consequently, facility) of a truck to arrive by road in an EU country from non EU countries. Number of roads crossing EU national boundaries with non EU states	FAO Geonetwork. Roads of the World
	P3t	Probability of returning trucks not being properly disinfected	[35]
	P4t	Potential ASF-contaminated waste introduced by cargo ships. Inward number of cargo ships from ASF-infected countries to EU ports	[25]-Traditional international trade database access (ComExt); [9]
	P5t	Potential ASF-contaminated waste introduced by passenger ships (excluding cruises). Inward number of passenger ships from ASF-infected countries to the EU	[25]-Traditional international trade database access (ComExt); [9]
	P6t	Potential ASF-contaminated waste introduced by Short sea shipping (SSS) movements. Ships from ASF-infected countries to the EU	[25]-Traditional international trade database access (ComExt); GIS
	P7t	Potential ASF-contaminated waste introduced by cruises. Proportion of cruise ships from ASF-affected areas per country	$P7t=\frac{\mathrm{CAi}}{\left[\mathrm{Cp}/p\right]}$
	CA	Number of cruise ships arriving at EU ports after one stop in ASF-infected areas	Travelocity. http://travel.travelocity.com
	Cp	Number of cruise passengers arriving at EU ports (Cp)	[25] (ComExt)
	p	Average number of passengers per cruise ship	Truecruises. http://www.truecruise.com/cruise-ship-database.aspx
	P8t	Potential contaminated waste introduced by international passenger flights. Commercial passenger flights from ASF-infected countries to EU airports	[25,9]
WB	P1_W_	Probability of wild boar becoming infected in country o through contact with infected wild boar	P1_w_ = P4_w_*P5 _w_
	P2_W_	Probability of wild boar becoming infected in country o through contact with infected domestic pigs	P2 _w_ = P6 _w_ *P7 _w_
	P3_W_	Probability of infected wild boar crossing national border	P3a _w_: P8 _w_ *P9 _w_ P3b _w_: P9 _w_ *P10 _w_
	P4_W_	Wild boar outbreak density in countries o	[9] 2007-2012
	P5_W_	Wild boar population density in countries o	[36-38]
	P6_W_	Density of domestic pig outbreaks in countries o	[9] 2007-2012
	P7_W_	Domestic pig population density in countries o	[36]
	P8_W_	Surface of shared wild boar suitable habitat along national borders	Corine land cover
	P9_W_	Distance from EU countries to the nearest outbreak (wild boar)	[9] 2007-2012
	P10_W_	Distance from EU countries to the nearest outbreak (domestic pig)	[9] 2007-2012

L.PIGS (Legal imports of pigs), L.PROD (Legal imports of products), ILLEGAL (Illegal imports), TAF (Transport associated fomites) and WB (wild boar).

#### Module 4: transport fomites

Similarly to the model for illegal imports, a proxy-based semi-quantitative model was used to estimate the risk of introduction through fomites associated with transport vehicles. The three routes that considered transport-associated fomites were: trucks returning from ASF-affected areas, waste from different ship types, and waste from international planes [21].

#### Module 5: wild boar movement

The risk of ASFV introduction through wild boar (*Sus scrofa L.*) movements was estimated for those EU countries sharing borders with non EU countries from Eastern Europe, considered to be the origin of the risk. For this purpose, a semi-quantitative model was employed that considered two sources of infection: wild boar infection in the country of origin through contact with ASF-infected wildlife (wild, feral or captive) or through contact with ASF-infected domestic pigs. Afterward, the introduction of ASFV into the EU was assumed to occur through the natural movement of infected wild boars or through contacts of infected wild boars with other wild boars. The detailed structure and parameters considered in the model are described in Table 1 and elsewhere [22].

### Overall risk of entry

The modular framework was implemented in Microsoft Excel (Microsoft Office 2010 Professional Edition) and is available as an additional file (Additional file 1). All the modules summarized in Section “Description of pathway modules” were included with a first worksheet that documented the methodology, module structure, assumptions and limitations, and a second worksheet with all the data inputs and calculations employed. A summary table with all the information (definitions and data sources) of all the parameters used in the framework is presented in Table 1. Additionally, the final risk scores of each country by all analyzed pathways were summarized in Table 2.

**Table 2:** Risk scores of the five modules for the 27 EU state members

**Country**	**Legal pigs**	**Legal products**	**Illegal**	**Transport**	**Wild boar**
Austria	0	0	2	1	NS
Belgium	1	0	1	2	NS
Bulgaria	0	5	2	2	2
Cyprus	0	0	2	1	NS
Czech Republic	0	0	2	1	NS
Denmark	1	1	1	2	NS
Estonia	2	0	2	3	3
Finland	4	0	2	3	5
France	4	3	4	2	NS
Germany	3	4	4	3	NS
Greece	4	0	2	2	1
Hungary	0	0	1	2	2
Ireland	2	2	1	1	NS
Italy	0	2	4	2	NS
Latvia	0	0	2	2	4
Lithuania	0	0	2	4	3
Luxembourg	0	0	1	1	NS
Malta	0	0	1	1	NS
Netherlands	0	1	2	2	NS
Poland	3	1	2	4	4
Portugal	0	0	2	2	NS
Romania	0	4	2	2	4
Slovakia	0	0	1	2	2
Slovenia	5	0	1	2	NS
Spain	0	1	3	2	NS
Sweden	5	0	2	2	NS
United Kingdom	3	3	4	2	NS

(NS: not studied). Risk scores equal or higher than 4 were highlighted using boldface numbers.

### Sensitivity analysis

For each module, sensitivity analyses were previously conducted to assess the robustness of the model and the influence of the different input parameters and/or the weights assigned to the proxies on the model results [18-22]. For the modular framework, the influence of the method used in each module to categorize the data of the proxies and risk scores was also assessed. In the individual risk assessments, categorization into risk scores was based on the natural breaks calculated with Jenks algorithm [24], except for the illegal pathway module. In the illegal pathway module, the parameter values tended to be clustered at lower values with a few extreme high values. Thus, Jenks algorithm led to quite low values which fell into relatively high risk scores. Therefore, a manual definition of the natural categories was preferred.

In the sensitivity analysis, other commonly used categorizing methods, specifically quantiles and geometric intervals, and Jenks algorithm for the illegal pathway, were used to calculate the risk scores for all five pathways. The results obtained with these alternative categorization methods were compared with the original method. Specifically, the list of countries at higher risk (a risk score equal to 4 and/or 5) for each pathway when using the different categorization methods is presented in descending order (from highest to lowest) in Table 3. Variations in the prioritization order, and the number of countries at higher risk, are also included in this table.

**Table 3:** Ordered list of country at highest risk per pathway using different categorization methods (NB: Jenks Natural Breaks, Q: Quantiles, GI: Geometric Interval; MNB: Manual Natural Breaks; RS: Risk Score)

**Pathway**	**Categorization method**	**Countries with RS 5**	**Countries with RS 4**	**Countries that underwent changes in risk order**	**New countries with RS 5**	**New countries with RS 4**
**Legal pigs**	**NB**	Sweden > Slovenia	Finland > Greece > France			
	**Q**	Sweden > Slovenia	Finland > Greece > France > Poland	0	0	1
	**GI**	Sweden > Slovenia	Finland > Greece > France > Poland > UK > Germany	0	0	4
**Legal products**	**NB**	Bulgaria	Romania > Germany			
	**Q**	Bulgaria > Romania > Germany > UK > France	Ireland > Italy > Netherlands > Spain	0	4	4
	**GI**	Bulgaria > Romania > Germany > UK > France	Ireland > Italy > Netherlands > Spain > Poland > Denmark	0	4	6
**Illegal**	**NB**	Italy > UK > Germany	Spain > France		3	
	**Q**	Italy > UK > Germany	Spain > France > Greece	0	3	1
	**GI**	Italy > UK > Germany	Spain > France > Greece > Finland > Sweden	0	3	3
	**MNB**	-	UK > Germany > France > Italy	2	−3	−1
**TAF**	**NB**	-	Poland > Lithuania			
	**Q**	-	Poland > Finland > Lithuania	1	0	1
	**GI**	-	Poland > Finland > Lithuania	1	0	1
**Wild boar**	**NB**	Finland	Romania > Latvia > Poland			
	**Q**	Finland > Romania	Latvia > Poland	0	1	−1
	**GI**	Finland > Romania	Latvia	0	1	−2

A sensitivity index was also calculated to assess the magnitude of the influence of the categorization methods on each module results. This sensitivity index (SI) was computed after considering the number of countries for which the risk score changed “CC”, the extent of this change “EC” (e.g., from 2 to 5, three levels of change), and the total chances of change for this pathway “TC”. This TC represents the sum of the potential levels of change for all the countries by this pathway (e.g., a country with an initial risk score of 3 has a maximum of three possible levels of change, from 3 to 0, whereas a country with a risk score of 1 has up to four possible levels of change). The sum of the possibilities for all the countries composed the TC for this pathway. The SI was computed as follows: $\mathrm{SI}=\frac{\sum \mathrm{LC}*\mathrm{CC}}{\mathrm{TC}}$

## Results

The model and its results are available as an Excel file in a public domain (www. asfrisk.eu) and as Additional file 1. The risk scores of ASFV introduction by the five pathways into each EU member state are presented in the results column on each pathway spreadsheet, and are also summarized in Table 2 and in the “overall results summary” worksheet of Additional file 1.

**Figure 3:**
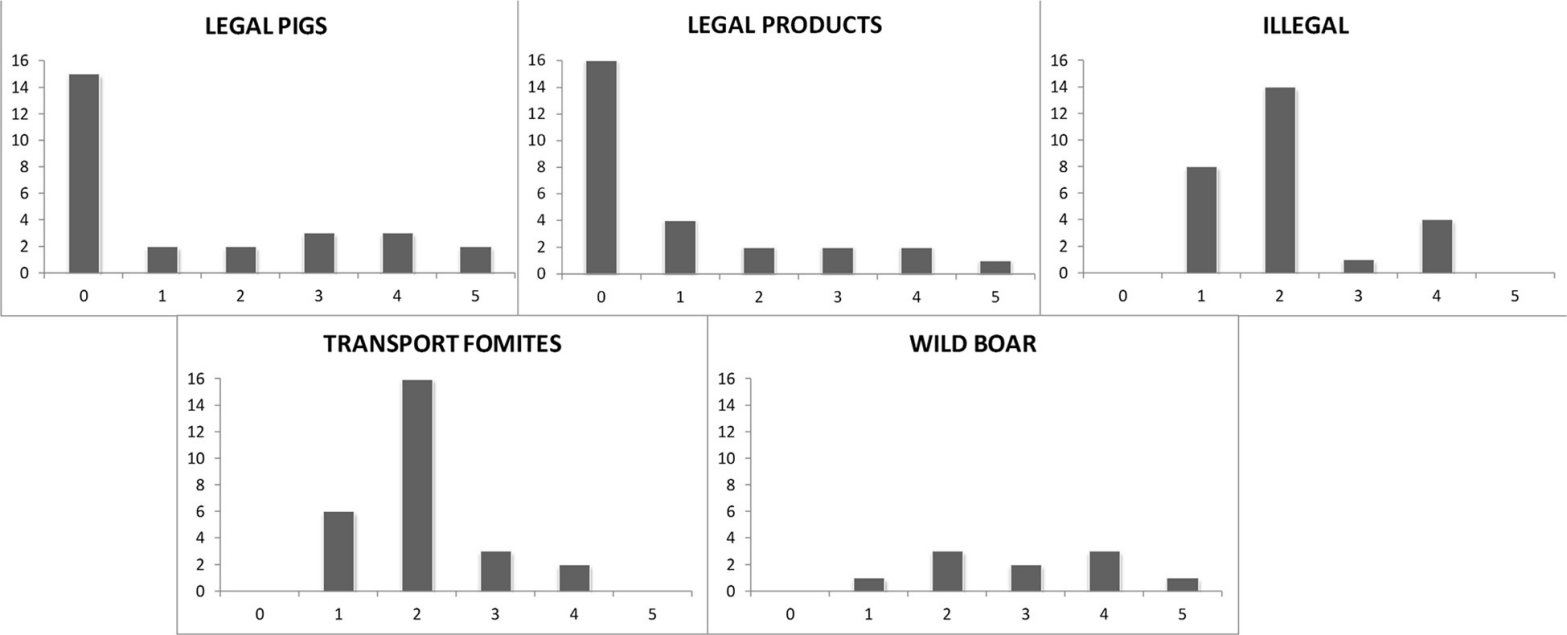
**Distribution of risk scores per pathway.** The number of countries per risk score was represented for the five assessed pathways.

The list of countries at higher risk (risk scores equal to 4 or 5) per pathway is provided in Table 2 in descending order, from highest to lowest risk (the natural breaks row). These are the countries that would benefit from prevention activities for each analyzed pathway. The average number of countries with high-risk scores per pathway was 3.6, and there were some pathways with only two countries at higher risk (transport fomites), whereas others had five (legal pigs) or four (illegal and wild boar) countries at higher risk.Different distributions of risk scores were observed between pathways (Figure 3). Whereas most countries presented a risk score of 0 (legal pathways) in some analyzed pathways, the majority of countries presented a risk score of 2 in others (illegal imports and transport fomites). Interestingly for these last two pathways, none of the countries presented the highest risk score of 5.Fifteen EU member states (55.5%) had at least one risk pathway with a risk score equal to or higher than 3 (Figure 3). Thirteen countries (48%) presented one risk score of 4 or 5. Four countries had a risk score of 5 for one pathway: Bulgaria for legally imported products during the HRP; Finland for wild boar, Slovenia and Sweden for legally imported pigs during the HRP. However, these countries did not necessarily obtain high risk scores in other pathways; indeed some (Bulgaria, Slovenia and Sweden) obtained risk scores of 0 and 1 for other pathways.

Among the 12 countries with lower risk scores (all risk scores lower than 3), some countries presented similar risk profiles, with the same risk scores for the same pathways.

### Sensitivity analysis

The results compiled in Table 3 reflect that for all the pathways and categorization methods, only two pathways presented differences in the order of countries at higher risk. In the TAF pathway, Lithuania and Finland interchanged their positions from the second to the third, and vice versa, when applying quantiles and geometric intervals instead of Jenks NB. For the illegal pathway, modifications in the order were observed when comparing Manual NB (the reference method) with the other categorization methods (Jenks NB, quantiles, geometric interval). Italy became the first country at risk (instead of forth by manual NB), and France came fifth (instead of third by manual NB) when using other methods. Consequently, the positions of the other countries changed, but not their relative order. For the other pathways, the only observed changes related to the inclusion of countries in category 5 (the countries considered at risk 4), or to those included in category 4.

The SIs calculated by the pathway and categorization method are presented in Table 4. The pathway that underwent the greatest modifications in terms of extent of change (EC), unlike the others, is that associated with legal import of products, which presented an average SI over all categorization methods evaluated at 34. This was also the only pathway for which some countries (8) underwent the maximum EC at three levels. In the remaining pathways, risk scores were less sensitive, with an SI of 8 (Legal imports of pigs and Transport pathways), 11 for the wild boar module and 12 for Illegal transport. The maximum EC observed in the other pathways was 2 levels, which affected four countries (legal imports of pigs) and one country in the illegal pathway. In the TAF and wild boar pathways, the countries’ risk scores changed one level at the most.

**Table 4:** Sensitivity indices obtained in the sensitivity analysis of each pathway and categorization method

	**SI quantiles**	**SI geometric Interval**	**SI manual natural breaks**	**Average SI per pathway**	**Average EC**	**Maximum EC (CC)**
**L. PIGS**	10.9	4.2	NA	7.6	1.2	2(4)
**L.PRODS**	31.4	35.5	NA	33.5	2	3(8)
**Illegal**	9.5	13.7	12.6	11.6	1.1	2(1)
**TAF**	5.6	10.1	NA	7.9	1.0	1(9)
**WB**	5.6	16.7	NA	11.1	1.1	1(6)

SI (Sensitivity Index), EC (Extent of change), CC (Number of countries that suffered this change); L.PIGS (Legal imports of pigs), L.PROD (Legal imports of products), ILLEGAL (Illegal imports of products), TAF (Transport associated fomites) and WB (wild boar); NA (Not applicable).

## Discussion

The modular framework herein presented integrates the methodology and outputs from five pathway-specific risk assessments to produce specific risk profiles for ASFV routes of entry into the EU. All the modules used the best data available, which need to be consistent and homogenously collected across EU member states. Due to the limited availability of detailed data and the huge variability between EU member states in terms of pig production characteristics, trade, environmental and even socio-cultural features, the results generated by this modular risk assessment framework need to be interpreted with caution. Some of the EU databases used for model parameterization were not as complete and detailed as national ones [18]. It has to be stated that this may have resulted in under- or overestimations of risk scores for some countries, depending on the reliability of the data available per country in these generic databases. Data on wild boar density and abundance of backyard pig production were limited at the time of the analysis performance, and could definitely affect the model results and the next assessment steps (backyard data could especially affect the future exposure assessment) [21]. The incorporation of recent FAO data on wild boars and low biosecurity farm [39] or wild boar density models based on ecological parameters [40] may improve the analysis. However, as the unit of analysis of this framework is the national level, these data are not expected to influence the final analysis outcome, especially when we consider that the densities of wild boars or domestic pigs in the individual sensitivity analysis of the wild boar pathway were the risk factor with the lowest influence on the model’s results [22].

The present framework integrates the calculations and results of five risk analyses using diverse methodological approaches for risk estimation. Legal import pathways were estimated by using stochastic quantitative models; consequently, an absolute probability of the risk of ASFV entry was obtained per country (available in Additional file 1). Only for this particular case can we state that the risk of ASFV introduction through legal imports of products during the HRP was higher than by legal imports of pigs during the same period. The probability of ASFV introduction through legal imports of pigs during the HRP was extremely low (the maximum probability was 0.000393 estimated for Sweden which, on average, corresponded to one introduction every 2544 years), while for the legal imports of products during the HRP it was much higher, with a maximum probability of 0.226 for Bulgaria (one expected introduction 4.4 years on average). These probabilities referred only to the risk of ASFV being released into the EU by these routes, without considering the probability of exposure, which could certainly modify the final risk of ASF outbreak occurring in the country. However, as the main goal of this work was to develop a method to integrate different types of model (fitting different data types and qualities) into the same framework, the probabilistic results were transformed into risk scores from 0 to 5, and took the same format as the other pathways. Nevertheless, both the detailed risk probabilities and any additional information can easily be consulted in the modular framework provided (see Additional file 1).

For the other pathways, estimated semi-quantitatively, it was not possible to make comparisons between routes, but between countries within the same route. The methodology selected for the semi-quantitative assessments differed substantially from the conventional methods, which combined matrices of numbers and risk terms. Risk matrices can provide an impression of higher accuracy and/or confidence if compared with qualitative assessments, which can be particularly wrong when scores are assigned and combined arbitrarily, and with no transparency [41]. In contrast, the systematic approach used herein produced risk scores by the weighted linear combination of the selected parameters contributing to the risk. The structure of these calculations, data inputs and weights used in the framework are presented in Additional file 1, thus ensuring the transparency of the model methods and results.

Nonetheless, as with any risk assessment model, a certain level of subjectivity was involved in selecting the pathways analyzed (based on the literature review on the ASFV transmission mechanisms, routes of introduction into previously free areas and the current epidemiological situation), module structure, methods applied, the parameters used as risk indicators (based on data availability), and categorization and weighting methods (analyzed in the sensitivity analysis). Attempts were made to minimize these limitations by means of the model’s transparency and the systematic application of the chosen approaches, which occurred with data categorization. After testing several categorization methods, natural breaks were used as this is the method that best adapts to the different distributions presented for all the input data. Within this method, the optimization method using Jenks algorithm was employed for most pathways to minimize intra-class variance and to maximize inter-class variances [24]. For the illegal import pathway however, the very skewed distribution of some parameter values led to relatively high-risk scores when Jenks adjustment was utilized, and it was thus decided to use manual natural breaks for this module. This observation is confirmed when comparing the list of countries at higher risk by each categorization method within illegal pathway (Table 2). The use of Manual NB provides a maximum risk score of 4 for four countries, whereas with Jenks NB three countries presented a risk score of 5. The lists of countries at higher risk observed among the two NB categorization methods were very similar, with two countries that modify their order and consequently change the position of the others but not their relative risk. Also the magnitude of the changes was not high (SI: 12.6) when comparing with other pathways. Consequently, although the magnitude of changes observed was not big, authors decided to employ Manual NB for this pathway for calculating the final results, as it better fits to the distribution of input data used and provides more adjusted results.

The sensitivity analysis assessed the impact of the categorization methods on the model’s outcomes and suggested satisfactory model robustness for all the pathways, except for the legal imports of products, which presented the greatest EC, with an average SI of 34. In addition, a maximum extent of change of 3 levels took place for eight countries for the legal imports of products pathway when modifying categorization. Within this pathway, data categorization was used only for transforming risk probabilities into risk scores, but conferred no additional value to the calculations (see the model structure in Figure 1). Consequently for this specific case, evaluating the absolute probabilistic values presented in Additional file 1 is recommended to better interpret the results instead of risk scores.

For the remaining pathways, the variations found by using other categorization methods were much slighter (SI less than 12), with a maximum extent of 2 levels of risk (legal imports of pigs during the HRP and illegal imports) and 1 (transport fomites and wild boar). This confirms the robustness of the model structure, especially for the semi-quantitative pathways where data categorization proved an important element to assess risk.

Besides the analysis of the EC observed (SI), the comparison of the main study output, specifically the list of countries at highest risk per pathway, revealed good consistency among the different categorization methods employed. Indeed the order of countries at highest risk changed only for the illegal pathway (when using manual NB, as previously discussed) and for TAF (when quantiles or geometric intervals were employed). Specifically for TAF, the values of two countries interchanged from second and third positions at risk, but both remained in the same risk category. For all the other pathways, no changes were observed in the list of the highest risk countries, except for the inclusion of some countries at the end of the list. These results suggest that the main model’s outputs were not influenced when other categorizing methods were used.

All five modules within the framework provided relative risk scores on a scale of risk scores from 0 to 5, the equivalent to risk probabilities from negligible to very high. Despite the use of different methodologies for each pathway module inhibits a direct comparison of the results among the pathways, as the same methodology was used for all the countries within each module, it was possible to compare the countries’ risk scores for a given pathway. Accordingly, these results can be used at the EU level to determine the countries/areas at higher risk per route of introduction. The application of specific control measures (adapted to the pathway’s origin of this risk) in these high-risk countries/pathways could prove beneficial for the whole EU. Based on this assumption, special attention should be paid to Sweden and Slovenia (risk of 5), followed by Finland, France and Greece (4), for the legal import of pigs pathway during the HRP. In relation with legal imports of products during the HRP, the only country at high risk was Bulgaria (5), followed by Romania and Germany (4). For illegal imports, no country presented the highest score (5), but a risk score of 4 was estimated for France, Germany, Italy and the United Kingdom. Something similar occurred with the transport associated fomites pathway, for which Lithuania and Poland obtained the highest scores (4). Finally, the risk deriving from the wild boar movement route was estimated with a score of 5 for Finland, followed by Latvia, Poland and Romania with a risk score of 4.

Some countries, like Finland, Romania, Germany, Poland and France, obtained risk scores of 4 and/or 5 for several pathways. These countries would benefit from further national research to elucidate which pathways are at higher risk for their country and which actions can be implemented to prevent these risks. Countries with similar risk profiles are also often similar in terms of external trade, geographic location, wild pig movements, and other factors that have been considered in the assessment. Germany and the UK obtained high risk scores for illegal imports, legal import and transport, which reflect their vast volume of trade with third countries [27]. They operate more flights from ASF-affected countries, and they also obtained the largest number of residents from ASF-affected countries, and very high values for inbound and outbound tourism (EUROSTAT data). In comparison to other EU countries, these two countries maintain very close relations with non EU countries, which could potentially incur a risk for disease introduction.

Transport and wild boar obtained risk scores of 4 or 5 in Lithuania, Finland or Poland, which reflects their geographical proximity to affected areas and, consequently, the facility of entrance by these routes influenced by distance. These results have been recently validated by the notification of ASF cases in dead wild boar in territories of Lithuania and Poland [9]. Malta and Luxembourg, with almost no pigs and an insignificant number of imports and risk relations, were at extremely low risk (around 0) for all the analyzed pathways.

In addition to these logical and biological arguments, the results obtained in the present framework agree with previous country-specific risk assessments. In a previous risk assessment for Finland, introduction by wild boar was considered to be the riskiest pathway [13], which agrees with the risk score of 5 obtained in the wild boar module of the present framework. Similarly, Germany obtained a risk score of 3 in the present transport fomites module and a moderate-high risk for the risk of the means of transport of pigs in the national assessment made by FLI [14]. These examples stress that despite the framework’s limitations, the obtained results agree with other assessments. This, consequently, inspires confidence in our model’s results. However, the authors are aware that these agreements do not guarantee the exact prediction of risk.

The analysis of the present results per pathway also provides interesting information on risk management. In some of the pathways analyzed, risk concentrated mainly in very few countries (transport fomites in two countries), whereas other pathways proved relevant for many countries (legal imports of pigs, illegal imports and wild boar). These findings suggest the benefits of a coordinated EU program for preventing and controlling the disease.

The outcomes obtained with this modular framework can be used to inform about the development of targeted risk management measures in the EU by implementing preventive measures in those pathways and countries that obtained higher risk scores. Additionally, the modular framework provided in the additional file can be used as a template to estimate the risk of ASFV introduction into other geographic areas or timeframes as more data become available.

## Conclusions

A modular framework has been implemented to estimate the risk of ASFV entry into the EU through five different introduction routes: the legal imports of pigs and products during the high risk period, the illegal imports of products, the transport associated fomites and the movement of wild boar. The framework, available in a public domain, integrates the five risk assessment modules and offers a transparent, easy-to-interpret tool that can be easily updated as data become available and can also be adapted to other regions of interest. The model’s results identify the EU countries at higher risk per route of ASFV introduction, and acts as a useful basis to develop a coordinated EU program and to, ultimately, improve ASFV prevention in the EU.

## Endnote

^a^Croatia was the EU country not included in the analysis given its recent inclusion in the EU (1/7/2013).

## Competing interest

The authors declare that they have no competing interests.

## Authors’ contribution

This work is the result of the long-term coordinated efforts made between three institutions (RVC, CISA-INIA and UCM). LM, BML, MM and JMSV carried out the analysis for the legal imports and transport fomites pathways. SC, BJ, FSV, DUP and BW made the illegal assessment. AT, MM and MJR developed the assessment for the wild boar pathway. The respective teams did the sensitivity analyses in the corresponding pathways (varying the categorization methods) and adapted their pathway analysis in order to integrate a common structure. LM integrated the modules into the common structure and wrote the draft of this manuscript. All the authors participated in the discussions about the selection of the methodologies used, the interpretation of the results, and the preparation of the manuscript by reviewing the results, and by notably modifying and suggesting changes to improve the study. All authors read and approved the final manuscript.

## Supplementary Material

Additional file 1 **Modular framework for estimating the risk of ASF introduction into European Union.** The present file includes input data, calculations and results of the five pathways assessed in the study. In addition, explanations about the structure and source of data were also included in separate sheets. Click here for file
